# Countering AI-generated misinformation with pre-emptive source discreditation and debunking

**DOI:** 10.1098/rsos.242148

**Published:** 2025-06-25

**Authors:** Emily R. Spearing, Constantina I. Gile, Amy L. Fogwill, Toby Prike, Briony Swire-Thompson, Stephan Lewandowsky, Ullrich K. H. Ecker

**Affiliations:** ^1^ School of Psychological Science, The University of Western Australia, Perth, Western Australia, Australia; ^2^ School of Psychology, The University of Adelaide, Adelaide, South Australia, Australia; ^3^ College of Social Sciences and Humanities, Northeastern University, Boston, MA, USA; ^4^ School of Psychological Science, University of Bristol, Bristol, UK; ^5^ Department of Psychology, University of Potsdam, Potsdam, Brandenburg, Germany; ^6^ Public Policy Institute, The University of Western Australia, Perth, Western Australia, Australia

**Keywords:** misinformation, generative artificial intelligence, source credibility, continued influence effect

## Abstract

Despite widespread concerns over AI-generated misinformation, its impact on people’s reasoning and the effectiveness of countermeasures remain unclear. This study examined whether a pre-emptive, source-focused inoculation—designed to lower trust in AI-generated information—could reduce its influence on reasoning. This approach was compared with a retroactive, content-focused debunking, as well as a simple disclaimer that AI-generated information may be misleading, as often seen on real-world platforms. Additionally, the extent to which trust in AI-generated information is malleable was also tested with an intervention designed to boost trust. Across two experiments (total *N* = 1223), a misleading AI-generated article influenced reasoning regardless of its alleged source (human or AI). In both experiments, the inoculation reduced general trust in AI-generated information, but did not significantly reduce the misleading article’s specific influence on reasoning. The additional trust-boosting and disclaimer interventions used in Experiment 1 also had no impact. By contrast, debunking of misinformation in Experiment 2 effectively reduced its impact, although only a combination of inoculation and debunking eliminated misinformation influence entirely. Findings demonstrate that generative AI can be a persuasive source of misinformation, potentially requiring multiple countermeasures to negate its effects.

## 1. Introduction

The capabilities of generative artificial-intelligence (AI) systems have grown exponentially in recent years. However, even the best and most widely used AI systems make errors. Generative AI models that specialize in text production—so-called large language models (LLMs; e.g. ChatGPT)—not only produce responses that may reflect the human biases present in the data on which they are trained, but often also fabricate information, a phenomenon known as ‘hallucinations’ [1–3]. The tendency for LLMs to produce misleading content, combined with the increasing accessibility of AI systems, has given rise to concerns that people may inadvertently consume and believe AI-generated misinformation, and that nefarious actors may use such systems to create misinformation at scale, with negative consequences for users and society [4,5].

It is clear from previous research that misinformation can have detrimental impacts, contributing to outcomes such as increased vaccine hesitancy and reduced satisfaction with democracy [6,7]; for a review, see [8]. Despite growing concerns that AI-generated misinformation may amplify these adverse outcomes, few studies have examined how it impacts people’s cognition. In the current study, we examined whether the impact of a misleading AI-generated article depends on its perceived source (human versus AI), and whether a pre-emptive, source-focused inoculation or a retroactive, content-based debunking can reduce the impact of AI-generated misinformation.

Generative AI tools are now commonplace. In 2024, ChatGPT has accumulated over 200 million active weekly users [9] and 65% of businesses now report regularly using generative AI systems [10], with user numbers approximately doubling in the past year. Despite having more regular interaction with AI systems, research suggests that people struggle to discriminate between AI-generated and human-generated content [11–14]. For example, Heppell *et al*. [4] found that participants were only 59% accurate at detecting AI-generated misinformation about the Ukraine war, and tended to overpredict human authorship. AI-generated misinformation may not only be hard to detect but may also be more compelling than human-generated misinformation, particularly when people are unaware of the origin of the information [15–18]. One recent study found that participants assessed the veracity of true AI-generated tweets more quickly and accurately than human-generated tweets, but were worse at identifying false information in AI-generated tweets than in human-generated tweets [19]. In other words, people were more often deceived by misinformation that was generated by AI than by misinformation that was generated by a person. One explanation for this finding is that LLMs can generate text that is easier and quicker to read than text written by humans, and the ease of processing this information is mistaken for a sign that the information is accurate [20,21].

Amid the growing popularity of generative AI systems and concerns about the inaccuracies in the output of such systems, some social media platforms have implemented warning labels to alert users to AI-generated content [22,23]. However, simply labelling AI-generated content may not be an effective countermeasure and may induce general scepticism [24]. For example, in some studies participants perceived headlines labelled as AI-generated to be less accurate and reported less intention to share them compared with the same headlines labelled as human-generated, regardless of headline veracity [25,26]. At the same time, generative AI platforms such as ChatGPT feature simple disclaimers that warn that AI-generated content can be inaccurate, but recent work suggests that these, too, are likely to have limited practical value [27]. For example, Kreps *et al*. [28] found that disclaimers warning that an article might contain misleading information and AI-generated content did not have a consistent effect; for Democrats, the disclaimer significantly reduced the perceived credibility of a politically congenial story but did not affect the perceived credibility of a politically non-congenial story, whereas the opposite was found for Republicans. Together, these findings suggest that neither labelling AI-generated content nor warning people about its potential inaccuracies by means of a simple disclaimer will be sufficient to reduce reliance on AI-generated misinformation.

Two strategies that may be more effective at reducing reliance on AI-generated misinformation are inoculation and debunking [29,30]. Inoculation involves pre-emptively warning people about an impending deception and providing an explanation of the misleading persuasive techniques that might be used [31,32]. Debunking interventions involve retroactively correcting misinformation after it has been encountered [33,34]. It is clear from numerous studies that both inoculation and debunking interventions can be effective at reducing misinformation reliance. The interventions can improve people’s ability to discern factual from misleading content, reduce people’s references to misinformation, and make people less likely to share misleading information [30,35,36]. It is also clear, however, that such interventions generally do not eliminate misinformation impacts entirely, with a large literature demonstrating that people frequently continue to rely on misinformation to some extent after having received clear and credible corrective interventions—a phenomenon known as the continued influence effect [29,37–39]. There is also little consensus on which intervention is the most effective for combatting misinformation influence. Some studies suggest that pre-emptive interventions are more effective because they enable people to develop counterarguments before encountering the misinformation, reducing its persuasiveness [40,41]. Conversely, other studies suggest that retroactive interventions are slightly more effective because both the misinformation and corrective information are accessible at the time of the intervention, making it easier to develop a coherent mental model [33,35,42–44]. By and large, however, inoculation and debunking approaches seem to be similarly effective for combatting misinformation influence (for a review, see [29,30]). This conclusion aligns well with findings from other areas, such as the comparable impact of pre-emptive and retroactive interventions on psychological reactance (e.g. in the context of health campaigns [45]).

One recommended—but rarely used—strategy for combatting misinformation is to discredit sources of biased or inaccurate information [46–48]. Source discreditation can reduce misinformation reliance by providing reasons for why the misinformation was initially presented and why it should be dismissed (e.g. the source tried to manipulate recipients due to a hidden agenda, or the source does not have the expertise to present accurate information on a topic). This idea is supported by theoretical models of misinformation influence that emphasize the weighting of perceived information reliability [49], and in line with empirical findings that perceived source credibility influences both belief [50–52] and correction effectiveness [53–56].

While source discreditation has been combined with other interventions in some research using ‘optimized’ debunking interventions [57], few studies have directly examined the effectiveness of source discreditation. Ecker *et al*. [46] found that highlighting a source’s conflict of interest or poor track record of communication reduced reliance on misinformation both for human and media sources. Connor Desai & Reimers [58], however, found that corrections stating that misinformation resulted from intentional deception versus unintentional error were equally effective. To the best of our knowledge, no study has assessed the effectiveness of a pre-emptive source discreditation. In light of these mixed results and growing concerns over AI-generated misinformation, the primary aim of the current study was to examine the effectiveness of a source-focused inoculation for reducing reliance on AI-generated misinformation.

## 2. Experiment 1

Experiment 1 aimed to explore the extent to which people’s trust in AI-generated information is malleable, and how this affects the influence of misleading AI-generated information presented in the form of a biased article. Participants were randomly assigned to read a misleading AI-generated article attributed to an AI or human source, or a generic AI-generated article that did not contain any misleading information. If the misleading article was attributed to an AI source, it was accompanied by a pre-emptive trust boost, a pre-emptive inoculation that discredited generative AI systems, a retroactive disclaimer that warned that generative AI can make mistakes, or no intervention. The trust boost outlined the benefits of AI systems (e.g. that they have a wealth of information available). By contrast, the inoculation explained why AI-generated content can be misleading (e.g. because generative AI systems are trained on potentially biased human data and sometimes fabricate information), whereas the disclaimer merely warned that AI systems can make mistakes without providing any explanation. We measured people’s general trust in AI-generated information, as well as their reliance upon specific AI-generated misinformation via inferential-reasoning questions. Although these two constructs are presumably related, it should be noted that they are distinct; people who are relatively trusting of AI-generated information in general may show minimal or no reliance on a specific piece of AI-generated information, and vice versa [59]. That is, even someone highly distrustful of AI content in general may rely on AI-generated information if it is consistent with their prior beliefs [38,49]. Materials and data for both experiments are available at: https://osf.io/t2g3a/.

We hypothesized that AI-generated misinformation would significantly influence reasoning (H1), with the size of the effect depending on its alleged source (H2).^1^ We further hypothesized that a trust-boosting statement would increase trust in AI-generated information (H3a) and specific misinformation reliance (H3b), that a source-focused inoculation would reduce trust in AI-generated information (H4a) and specific misinformation reliance (H4b), and that a simple disclaimer would have no effect on trust in AI-generated information (H5a) or specific misinformation reliance (H5b); finally, we predicted that misinformation would continue to have some influence on reasoning post-interventions (H6). For a summary of supported and rejected hypotheses across both experiments, please see electronic supplementary material, table S1.

### 2.1. Method

Experiment 1 used a between-subjects design with six conditions: control (non-misleading article); human misinformation (misleading article with human byline); AI misinformation (misleading article with AI byline); trust boost (passage designed to boost trust in AI followed by the misleading article with AI byline); inoculation (source-focused inoculation passage followed by the misleading article with AI byline); or disclaimer (misleading article with AI byline followed by a disclaimer).

#### 2.1.1. Participants

An *a priori* power analysis conducted using G*Power 3.1 [60] indicated that at least 100 participants in each experimental condition were required to detect a small effect (*f* = 0.2, *α* = 0.05, and 1 – *β* = 0.80). We collected data from 631 English-speaking adults from the United States via Prolific (https://www.prolific.com/), using representative sampling.^2^ We excluded participants due to a self-reported lack of effort (*n* = 1) or inconsistent responding on the reasoning (*n* = 7) and trust (*n* = 23) measures, as per *a priori* exclusion criteria (see electronic supplementary material for details). The final sample comprised *n* = 603 participants, including 297 women, 295 men, 10 non-binary participants and 1 participant who self-described as transgender male. Age ranged from 18 to 80 years (*M* = 44.83, SD = 16.01). Participants were randomly allocated to one of the six conditions, with the constraint of approximately equal cell sizes.

#### 2.1.2. Materials

##### 2.1.2.1. Articles

The freely accessible version of ChatGPT v3.5 was used to generate a biased, 529-word article in favour of trickle-down economics, titled ‘The Case for Trickle-Down Economics: Fostering Prosperity for All’.^3^ The topic of trickle-down economics was chosen based on pilot-testing indicating that participants tend to be familiar with the concept without having detailed knowledge or a firm stance on the topic, and to avoid exposing participants to misinformation with a high potential for direct harm (e.g. health misinformation; [61]). ChatGPT was prompted to provide a biased, one-sided perspective, despite this being potentially misleading. The final article was based on two ChatGPT responses that were combined and edited to improve clarity and flow. This approach mimics how a malicious actor may use generative AI to produce effective misleading content. The article was identical in the human and AI-misinformation conditions, except that the header stated that the article was written by a human author (‘Alex Kennedy’) or ChatGPT, respectively. The source information was presented twice more, in bold font, in the survey instructions, to ensure participants noticed and encoded it.

In the control condition, participants were given an unbiased, 378-word article on the retirement of a fictional radio host, titled ‘A Decade of Voices: Reflecting on a Local Radio Legend’s Journey’.^4^ The article featured several quotes from the fictional radio host, Jim Morgan (e.g. he was quoted saying that ‘The world changes rapidly, the economy goes up and down, but what remains constant is the power of people’s stories’). The topic was chosen to be unrelated to the misleading article, while ensuring that the article remained sufficiently relevant to the questionnaires to avoid arousing suspicion or confusion about the study. To this end, the article mentioned that the radio host had often discussed topics related to economics, and included some titbits related to the economy.

### 2.1.3. Interventions

#### 2.1.3.1. Trust boost

In the trust-boost condition, participants read a brief 169-word passage outlining the advantages of AI-generated information. For example, ‘The algorithms that AI chatbots rely on have access to a wealth of human-generated data and knowledge, which they use to make predictions and generate information in real time’). The passage explained that AI companies have ‘put safeguards in place to ensure that AI systems do not produce information that may go beyond their capabilities’, which ‘ensures that AI-generated information can become increasingly accurate and trustworthy’.

#### 2.1.3.2. Source-focused inoculation

In the inoculation condition, participants read a 174-word passage designed to reduce trust in AI-generated information, which highlighted the potential for AI to generate biased or inaccurate information. For example, ‘Because AI tools are trained on large, human-generated datasets, AI-generated information can reflect human biases and stereotypes. In other words, AI systems are only as accurate as the human-generated data they are trained on. […] AI-generated information can also produce absurd claims and be outright false. This is because AI algorithms are unable to distinguish between accurate and inaccurate information when generating content.’

#### 2.1.3.3. Disclaimer

In the disclaimer condition, participants read a statement after the misleading article that pointed to the tendency of AI systems to make errors; this was the original disclaimer currently used by ChatGPT (i.e. ‘ChatGPT can make mistakes. Check important info.’).

#### 2.1.3.4. Questionnaires

Participants completed two questionnaires. First, to examine how much participants relied on misinformation in their reasoning, they were asked to rate their agreement with seven statements relating to trickle-down economics (e.g. ‘Reducing taxes for corporations and higher-income earners would ultimately benefit all of society’) on an 11-point scale ranging from 0 (*strongly disagree*) to 10 (*strongly agree*). Second, participants rated eight items regarding their level of trust in AI-generated misinformation (e.g. ‘I trust AI-generated content’) on the same 11-point scale. Each questionnaire contained two reverse-coded items.

### 2.1.4. Procedure

Participants viewed an ethics-approved information page and provided informed consent by ticking a box, before providing some demographic information (i.e. age, gender). Participants were told that the study was ‘investigating how we process information about current economic affairs’. Participants were then presented with the article and intervention text as per their assigned condition. Text was presented in paragraphs, each presented on a separate page. Reading was self-paced, but each page was presented for a minimum time (set at approximately 100 ms per word). Participants then completed a 1 min distractor task (a word puzzle), before responding to the inferential-reasoning and trust questionnaires. They were also asked to indicate whether they put in a ‘reasonable effort’ or whether their data should be discarded. Finally, participants were fully debriefed following best-practice guidelines [61] and compensated £1.50 (approx. US$1.90). The experiment took approximately 8−10 min.

### 2.2. Results

#### 2.2.1. Trust in AI-generated information

**Figure 1.**
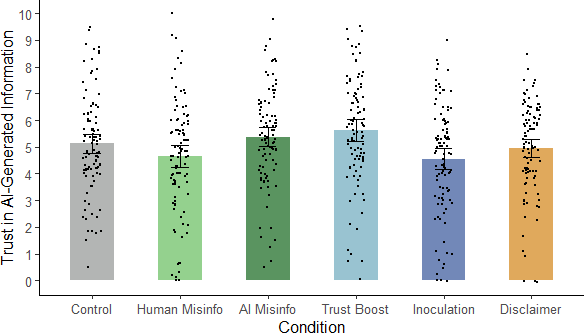
Mean trust in AI-generated information across conditions in Experiment 1. *Not*e: Misinfo., misinformation; error bars show 95% confidence intervals.

Before turning to the analysis of misinformation reliance, we first checked whether the interventions were effective at impacting trust in AI-generated information. To this end, we conducted a one-way ANOVA on trust scores, calculated by averaging participants’ responses to the trust questions (after reverse-scoring relevant items). Scores ranged from 0 to 10, with higher scores indicating greater trust. This analysis revealed a significant main effect of condition, *F*(5, 597) = 4.69, *p* < 0.001, η_p_^2^ = 0.038 (see figure 1).

To test specific hypotheses regarding the interventions’ influence on trust in AI-generated information, we conducted planned contrasts comparing each condition with the AI-misinformation condition, applying the Holm–Bonferroni correction for each set of contrasts in a hypothesis-specific manner (see table 1). The analyses revealed that the inoculation significantly reduced general trust in AI-generated information compared with the AI-misinformation condition, supporting H4a. Neither the trust boost nor the disclaimer significantly impacted trust in AI-generated information, by contrast to H3a but in line with H5a. To quantify evidence for the absence of effects, we conducted Bayesian independent-samples *t*-tests, which revealed moderate evidence for the null hypothesis in case of the trust boost (*BF*_01_ = 4.42) and anecdotal evidence for the null for the disclaimer (*BF*_01_ = 1.57).

**Table 1.** Planned contrasts on trust scores in Experiment 1.

hypothesis	contrast	*F*(1, 597)	*p*	η_p_^2^
H3a	AI misinfo. versus trust boost	0.79	0.373	0.001
H4a	AI misinfo. versus inoculation	9.45	<0.001*	0.016
H5a	AI misinfo. versus disclaimer	2.57	0.109	0.004

* indicates statistical significance after Holm–Bonferroni adjustment.

Misinfo., misinformation.

Additional exploratory contrasts were run to check whether exposure to the biased article itself affected trust (see electronic supplementary material, table S2). These contrasts suggested that neither the human-misinformation nor the AI-misinformation condition differed significantly from control, although trust scores were higher in the AI-misinformation condition than the human-misinformation condition.

#### 2.2.2. Misinformation reliance

**Figure 2.**
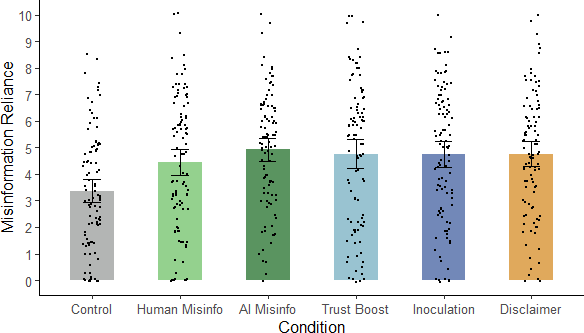
Mean misinformation reliance across conditions in Experiment 1. *Note*: Misinfo., misinformation; error bars show 95% confidence intervals.

For the main analyses of misinformation reliance, we calculated misinformation-reliance scores by averaging each participant’s responses to the inferential-reasoning questionnaire (after reverse-scoring relevant items); scores ranged from 0 to 10, with higher scores indicating greater misinformation reliance. A one-way ANOVA was then performed on these scores, which indicated a significant main effect of condition, *F*(5, 597) = 5.69, *p* < 0.001, η_p_^2^ = 0.046 (see figure 2).

To directly assess our specific hypotheses, we again conducted planned contrasts (see table 2). First, we tested whether the misleading article influenced participants’ reasoning. In support of H1, we found misinformation reliance was higher in both misinformation conditions than in the control condition. Second, we tested the effect of the alleged source on misinformation reliance. The prediction that misinformation impact would depend on the perceived source (H2) was not supported, with no significant difference between misinformation-reliance scores in human- and AI-misinformation conditions. Accordingly, a Bayesian independent-samples *t*‐test yielded anecdotal evidence for the null hypothesis (*BF*_01_ = 2.48). Third, we tested whether the interventions were effective at influencing misinformation reliance by comparing each intervention condition (trust boost, inoculation, disclaimer) with the AI-misinformation condition. All contrasts were nonsignificant, suggesting that none of the interventions significantly influenced misinformation reliance. H3b and H4b were thus not supported, although the null effect of the disclaimer was predicted (H5b). Bayesian independent-samples *t*-tests yielded moderate evidence for the null hypothesis across the trust-boost (*BF*_01_ = 5.85), inoculation (*BF*_01_ = 5.71) and disclaimer conditions (*BF*_01_ = 5.78). Finally, we compared the control condition with the inoculation and disclaimer conditions to test for continued influence. As expected, and in line with earlier comparisons, participants continued to rely on the AI-generated misinformation in their reasoning after receiving the interventions, in support of H6.

**Table 2.** Planned contrasts on misinformation-reliance scores in Experiment 1.

hypothesis	contrast	*F*(1, 597)	*p*	η_p_^2^
H1	control versus human misinfo.	10.04	0.002*	0.017
	control versus AI misinfo.	20.71	<0.001*	0.034
H2	human misinfo. versus AI misinfo.	1.97	0.161	0.003
H3b	AI misinfo. versus trust boost	0.22	0.639	<0.001
H4b	AI misinfo. versus inoculation	0.27	0.603	<0.001
H5b	AI misinfo. versus disclaimer	0.22	0.638	<0.001
H6	control versus inoculation	16.52	<0.001*	0.027
	control versus disclaimer	16.65	<0.001*	0.027

* indicates statistical significance after Holm–Bonferroni adjustment.

Misinfo., misinformation.

### 2.3. Discussion

In Experiment 1, we tested whether providing a trust boost or a source-focused inoculation could influence participants’ general trust in AI-generated information; whereas our trust boost was ineffective, we found that a source-focused inoculation significantly reduced trust in AI-generated information. We also examined whether AI-generated misinformation would influence participants’ reasoning, and whether this effect was dependent on the perceived source. The provided misinformation had the expected persistent influence on participants’ reasoning, replicating much previous research (see [29]). Misinformation impact was comparable regardless of the perceived source, suggesting that people are neither more likely nor less likely to believe and rely on information from an AI source than from an unfamiliar human source. No intervention was effective at reducing the effect of the misinformation on reasoning; participants were influenced by the misinformation regardless of whether they received an initial AI trust boost, a source-focused inoculation, a simple disclaimer or no intervention. The ineffectiveness of a disclaimer was not surprising given its generic nature and prior findings [28]. The ineffectiveness of the trust boost is also understandable given it had no impact on trust in AI-generated information to begin with. However, the ineffectiveness of the source-focused inoculation is noteworthy given that it did significantly reduce AI trust, and given that technique-based inoculations and retrospective source discreditations have been found to be effective in prior work [31,46,62,63].

One possible reason why the pre-emptive source-focused inoculation had no effect may be that people lacked sufficient knowledge to identify the flaws in the misleading arguments. Consistent with this idea, research informed by mental-model theory has suggested that interventions may fail to reduce misinformation reliance if they do not explain why the misinformation was wrong or how the information came to be [34,64,65]. If inoculated participants were more sceptical of AI-generated content but were unable to identify flaws in the biased argument, then it is possible that a pre-emptive source discreditation would be effective when accompanied by a retroactive debunking that provided some explanation regarding the falsity of the information provided. This was tested in Experiment 2.

## 3. Experiment 2

In Experiment 1, we found that a source-focused inoculation reduced trust in AI-generated information but did not significantly influence misinformation reliance. Experiment 2 further examined the efficacy of a pre-emptive source-focused inoculation to reduce misinformation reliance, and compared its effects with a retroactive, content-focused debunking. Whereas the disclaimer in Experiment 1 merely warned participants that generative AI systems ‘can make mistakes’, the debunking intervention in Experiment 2 explicitly told participants that the misleading article contained inaccurate content and highlighted criticisms of trickle-down economics. Although both pre-emptive and retroactive interventions have been found useful, it is still unclear which approach is more effective at combating the influence of misinformation [33,35,41,42]. The combination of (retroactive) source-focused and content-focused interventions has been found to be particularly effective, presumably because a combined intervention discredits both the misinformation itself and the source of that information [46].

In Experiment 2, participants again read a misleading AI-generated article about trickle-down economics, attributed to either a human or AI source (or a non-misleading generic article in a control condition). To ensure a debunking approach was applicable, ChatGPT was prompted to produce fabricated and misleading arguments that were incorporated into the misleading article. In the AI-misinformation conditions, the misleading article was presented with a pre-emptive, source-focused inoculation that discredited generative AI systems, a retroactive, content-focused debunking that identified the misleading nature of the AI-generated arguments, both an inoculation and a debunking, or no intervention. Misinformation reliance was again assessed by examining participants’ answers to inferential-reasoning questions.

We hypothesized that AI-generated misinformation would impact reasoning (H1) and that the size of this effect may depend on its alleged source (H2); that a source-focused inoculation would reduce trust in AI-generated information (H3a) and misinformation reliance (H3b); that a content-focused debunking would reduce misinformation reliance (H4); and that a combined intervention would reduce trust in AI-generated information (H5a) and misinformation reliance (H5b). We also expected that the combined intervention would produce a larger reduction in misinformation reliance than inoculation or debunking alone (H6), and that there would be continued influence of the misinformation on reasoning post-interventions (H7).^5^

### 3.1. Method

Experiment 2 used a between-subjects design with six conditions: control (non-misleading article); human misinformation (misleading article with human byline); AI misinformation (misleading article with AI byline); debunking (misleading article with AI byline followed by debunking); inoculation (source-focused inoculation followed by misleading article with AI byline); and combination (misleading article with AI byline sandwiched by source inoculation and debunking).

#### 3.1.1. Participants

As in Experiment 1, we aimed for data from 600 participants. Data were collected from 634 English-speaking adults from the United States via Prolific, again using representative sampling. We excluded participants due to a self-reported lack of effort (*n =* 1) and inconsistent responding on the reasoning (*n* = 4) and trust (*n* = 9) measures (see electronic supplementary material for details). The final sample comprised *n* = 620 participants, including 312 women, 299 men, 8 non-binary participants and 1 participant who self-described as gender-fluid. Age ranged from 18 to 86 (*M* = 44.85, SD = 15.74). Participants were randomly allocated to one of the six conditions, with the constraint of approximately equal cell sizes.

#### 3.1.2. Materials

##### 3.1.2.1. Articles

We crafted a new misleading article titled ‘The Benefits of Trickle-Down Economics’ by combining three generic and seven misleading statements relevant to trickle-down economics, which were again generated by ChatGPT. The generic statements were placed at the beginning of the article and provided introductory, unbiased information about economics (e.g. ‘Economics is a social science that examines how individuals, businesses, governments and societies make choices about allocating resources to satisfy their wants and needs’). For the misleading statements, ChatGPT was initially prompted to generate 20 statements that presented misleading or factually inaccurate information in support of trickle-down economics, including fabricated elements such as fake experts, statistics and quotes.^6^ For example, one misleading statement read: ‘Professor Michael Roberts’ research, presented at the International Conference on Economics and Innovation (ICEI), demonstrated the effectiveness of targeted tax incentives for technology startups. He revealed a 30% increase in innovation and patent filings, thereby fuelling technological progress and industry expansion’ (note that the expert, conference and evidence were all fabricated).

As in Experiment 1, some statements were edited to improve clarity and to mimic how a malicious actor might use generative AI to produce misinformation. We pilot-tested the initial 20 statements by asking a separate sample of *n* = 59 Prolific participants to rate each statement’s persuasiveness on an 11-point scale ranging from 0 (*not at all persuasive*) to 10 (*very persuasive*). We then selected the seven most persuasive statements for the misleading article. These were introduced in the article as ‘the top seven arguments for trickle-down economics’. The article had 366 words and again was presented either with a human (‘by Alex Kennedy’) or AI (‘by ChatGPT’) byline, respectively. Survey instructions again referred to the respective article source twice more, in bold font, to ensure participants encoded the source information. The control article comprised only the three generic statements (60 words).

### 3.1.3. Interventions

#### 3.1.3.1. Source-focused inoculation

The source-focused inoculation was presented in the format of a 301-word passage titled ‘The Limitations of AI Technology’. Like in Experiment 1, the inoculation article highlighted the risk of human biases and fabricated information in AI-generated output, but provided additional examples of situations where AI has been found to fabricate plausible yet inaccurate information and highlighted the potential for malicious actors to use AI to create misleading content. In particular, the passage used in this experiment described generative AI tools, before warning participants about the threat of misleading AI-generated information (e.g. ‘Users need to be mindful of the limitations of AI tools and that AI-generated information might mislead them’). The article then explained that generative AI tools can be prone to bias due to their training datasets (e.g. ‘AI systems tend to exhibit significant gender, racial and political biases’), and provide fabricated information such as fake evidence (e.g. ‘AI tools can also invent fake evidence—statistics, quotes or specific research studies—that may also be attached to a real or fake expert. This can create the illusion that the presented information is expert-endorsed and therefore credible and truthful, when in fact it is not based on factual or even existent evidence’). The article also explained that AI can be used by malicious actors to purposefully produce misleading arguments (e.g. ‘Malicious actors can easily create and spread such arguments quickly and widely to influence others for their own gain, or in pursuit of a hidden agenda. For example, a person with a vested interest may use an AI chatbot to generate misleading arguments about a particular topic, which they then disseminate through social media’).

#### 3.1.3.2. Debunking

The content-focused, 77-word debunking intervention informed participants that some of the arguments for trickle-down economics presented in the article had been ‘fact-checked and found to be misleading’, and that the evidence provided ‘was largely incorrect and presented trickle-down economics in an overly positive manner.’ A brief description of the criticisms aimed at trickle-down economics was also provided (e.g. ‘While the theory is supported by some, it has also been criticized for disproportionately benefitting the wealthy, not leading to substantial job creation, and providing limited economic growth’).

#### 3.1.3.3. Combined

The combined intervention comprised both the source-focused inoculation (presented before the misleading article) and the content-focused debunking (presented after the misleading article), which were identical to those in the other intervention conditions.

#### 3.1.3.4. Questionnaires

We used the same inferential-reasoning and trust questionnaires as in Experiment 1.

### 3.1.4. Procedure

The procedure was identical to Experiment 1. Participants received £1.35 (approximately US$1.56) for completing the experiment, which took approximately 8−10 min.

### 3.2. Results

#### 3.2.1. Trust in AI

**Figure 3.**
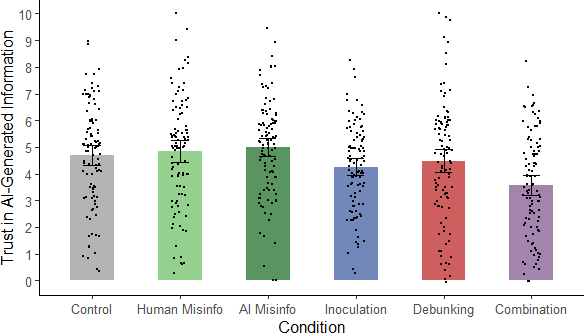
Mean trust in AI-generated information across conditions in Experiment 2. *Note*: Misinfo., misinformation; error bars show 95% confidence intervals.

To examine whether the interventions influenced participants’ general trust in AI-generated misinformation, a one-way ANOVA on trust scores was conducted, which revealed a significant main effect of condition, *F*(5, 614) = 6.97, *p* < 0.001, η_p_^2^ = 0.054 (see figure 3).

Planned contrasts were then run to test specific hypotheses (see table 3). First, to test whether inoculation was effective at reducing trust in AI-generated misinformation, inoculation and AI-misinformation conditions were compared. As expected, trust was lower in the inoculation condition, in support of H3a. Trust levels were also lower in the combined condition relative to the AI-misinformation condition, in line with H4a.

**Table 3.** Planned contrasts on trust scores in Experiment 2.

hypothesis	contrast	*F*(1, 614)	*p*	η_p_^2^
H3a	AI misinfo. versus inoculation	7.34	0.007*	0.012
H4a	AI misinfo. versus combination	26.89	<0.001*	0.042

* indicates statistical significance after Holm–Bonferroni adjustment.

Misinfo., misinformation.

Additional exploratory contrasts were again run (see electronic supplementary material, table S3). Although a content-focused debunking intervention was not expected to influence trust, it is nevertheless possible that it could affect the perceived trustworthiness of the AI source by demonstrating that AI can sometimes produce inaccurate information. However, there was no significant difference between the AI-misinformation and debunking conditions (although there was a significant difference between the inoculation and combination conditions, suggesting that the addition of the debunking further reduced trust relative to the inoculation alone). Finally, we checked whether merely being exposed to a misleading article affected trust. Again, neither the human- nor the AI-misinformation condition differed significantly from control, nor did the two misinformation conditions differ from each other.

#### 3.2.2. Misinformation reliance

**Figure 4.**
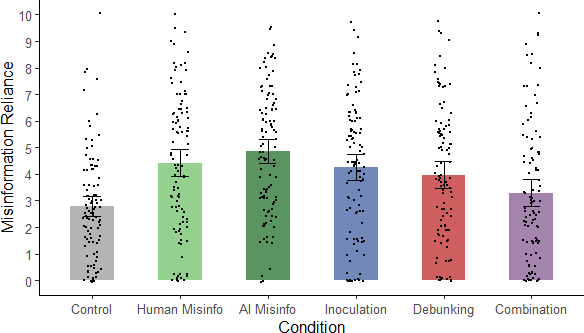
Mean misinformation reliance across conditions in Experiment 2. *Note*: Misinfo., misinformation; error bars show 95% confidence intervals.

To assess whether the interventions were effective at reducing misinformation reliance, a one-way ANOVA on misinformation-reliance scores was run, again yielding a significant main effect of condition, *F*(5, 614) = 10.12, *p* < 0.001, η_p_^2^ = 0.076 (see figure 4).

Next, we conducted planned contrasts to test our hypotheses (see table 4). First, the hypothesis that AI-generated misinformation would influence reasoning (H1) was supported, with greater scores in the two misinformation conditions relative to control. Second, we tested whether misinformation reliance differed between the human- and AI-misinformation conditions; the result was nonsignificant, so H2 was not supported. This was corroborated by a Bayesian independent-samples *t*‐test (*BF*_01_ = 3.10). Third, we tested whether the interventions were effective at reducing reliance on AI-generated misinformation. Misinformation-reliance scores did not differ significantly between AI-misinformation and inoculation conditions, so in replication of Experiment 1, H3b was not supported. A Bayesian independent-samples *t*‐test returned support for the null hypothesis, if only anecdotal (*BF*_01_ = 1.40). As predicted, the debunking and combined interventions did significantly reduce misinformation reliance relative to the AI-misinformation condition, supporting H4 and H5b. (It should be noted, however, that misinformation reliance scores in the debunking condition did not significantly differ from those in the inoculation condition, *F*(1, 614) = .69, *p* = .41, η_p_^2^ = 0.001; a Bayesian *t*‐test yielded *BF*_01_ = 4.90.) The combination condition was also associated with lower misinformation-reliance scores than either the inoculation or debunking conditions, in support of H6. Finally, to test for continued influence post-intervention, we compared the control condition separately with the three intervention conditions. As predicted, misinformation-reliance scores were higher in the inoculation and debunking conditions than control, demonstrating continued influence, in support of H7. However, there was no evidence for a continued influence effect in the combined condition, meaning that misinformation reliance in the combined condition was not statistically discernible from baseline; correspondingly, a Bayesian *t*‐test yielded *BF*_01_ = 2.11.

**Table 4.** Planned contrasts on misinformation-reliance scores in Experiment 2*.*

hypothesis	contrast	*F*(1, 614)	*p*	η_p_^2^
H1	control versus human misinfo.	22.65	<0.001*	0.036
	control versus AI misinfo.	37.51	<0.001*	0.058
H2	human misinfo. versus AI misinfo.	1.67	0.196	0.003
H3b	AI misinfo. versus inoculation	3.27	0.071	0.005
H4	AI misinfo. versus debunking	6.98	0.008*	0.011
H5b	AI misinfo. versus combination	21.5	<0.001*	0.034
H6	combination versus inoculation	7.93	0.005*	0.013
	combination versus debunking	3.97	0.047*	0.006
H7	control versus inoculation	18.44	<0.001*	0.029
	control versus debunking	12.05	<0.001*	0.019
	control versus combination	2.16	0.142	0.004

* indicates statistical significance after Holm–Bonferroni adjustment.

Misinfo., misinformation.

To put these findings into perspective, effect sizes for the inoculation (*d* = 0.25) and debunking (*d* = 0.36) interventions in this study were relatively modest compared with averages found in meta-analyses. In terms of inoculation, for example, Banas & Rains [66] found an average effect size of *d* = 0.43. In terms of debunking, Walter & Murphy [44] found that retroactive corrections generally had a large effect (*d* = 0.82) on misinformation belief, but corrective messages focusing on source credibility produced only a moderate effect (*d* = .28) more in line with our findings. Additionally, it should be noted that more detailed corrections are generally more effective at reducing misinformation influence, which may explain the relatively modest effect of our debunking intervention, given that it did not specifically target all the misleading arguments presented [67,68].

### 3.3. Discussion

Experiment 2 examined whether a pre-emptive source-focused inoculation, a retroactive debunking, or a combination of both interventions could reduce people’s reliance on AI-generated misinformation. Experiment 2 replicated the finding that AI-generated misinformation influenced participants’ reasoning, regardless of the perceived source. Also consistent with Experiment 1, discrediting AI as a source of reliable information pre-emptively through inoculation did not significantly reduce the subsequent impact of the specific misinformation provided, even though it again reduced participants’ general trust in AI-generated information. By contrast, a retroactive debunking that identified the provided article as misleading was effective at reducing misinformation reliance (although we again note that there was no statistical evidence that the two interventions’ impacts differed from each other). The superior efficacy of the combined intervention indicates that a source-focused inoculation was able to reduce misinformation reliance when it was supported by a subsequent debunking (or, alternatively, that debunking was more effective when the source had already been discredited).

## 4. General discussion

Across two experiments, we examined the effectiveness of a simple disclaimer, a pre-emptive source-focused inoculation, and a retroactive content-focused debunking to reduce reliance on AI-generated misinformation. We also explored whether a trust-boosting intervention would increase misinformation reliance. We found that AI-generated misinformation influenced people’s reasoning regardless of whether it was attributed to a human or AI source. A disclaimer had no impact on misinformation reliance, demonstrating again that generic, tentatively worded corrections are likely to have merely token value [69,70]. A trust boost was also inconsequential. Perhaps more surprisingly, a source-focused inoculation was similarly unable to reduce misinformation reliance, despite reducing general trust in AI-generated information. Our pre-emptive intervention differed substantively from the most common type of inoculation, which is technique- and not source-based [31,71,72]; whereas the aim of technique-focused inoculation is to provide people with the skills to detect misleading arguments by providing an explanation of the techniques that might be used to mislead, the aim of the source-focused inoculation was to reduce reliance on the misinformation by undermining the source’s credibility and explaining why it sometimes provides inaccurate information. However, this finding nevertheless demonstrates that not all pre-emptive inoculation-type interventions are effective or even superior to retroactive interventions [40,41]. In fact, in the present study, a retroactive debunking was able to significantly reduce the influence of misinformation on participants’ reasoning, in line with much previous research [33,35,44,73]. Only a combination of inoculation and debunking, however, was able to eliminate the influence of misinformation entirely [74,75].

Our finding that a pre-emptive source discreditation was largely ineffective at reducing misinformation reliance stands in contrast to previous work on retroactive source discreditation [46]. One possible explanation for this discrepancy is that, although inoculated participants in the present study were less trusting of AI-generated information in general, they did not have sufficient knowledge of trickle-down economics to counter the misleading arguments presented. Consistent with this idea, the literature on mental models suggests that misinformation countermeasures will be more effective when they provide sufficient information for participants to update their existing mental model with new evidence and to understand why the previously encountered information was wrong [34,64,65,76]. This interpretation is also supported by the finding that, although source inoculation alone did not reduce misinformation reliance, it nonetheless contributed to eliminating the influence of misinformation in the combination condition, where a debunking provided additional information to allow for updating that could preserve mental-model coherence.

If source-focused interventions are only effective at combating misinformation when people can generate counterarguments based on specific knowledge they have, then it follows that the effectiveness of source discreditation may generally depend on people’s level of knowledge about a topic. If people have no relevant background knowledge, they may be unable to identify the flaws in a misleading argument; if they have significant relevant expertise, they may be able to reject misinformation outright [77–79]. In both these situations, source credibility may have little to no effect on misinformation reliance. If people have moderate levels of knowledge about a topic, however, they may be able to generate counterarguments, but may not do so unless they are alerted to a potentially untrustworthy source. This may also explain the effectiveness of retroactive source discreditation in the study by Ecker *et al*. [8], which used misinformation in social scenarios that participants likely had some stereotypical knowledge of (e.g. reasons for a restaurant closure).

Another possible reason why source discreditation did not reduce misinformation reliance in this study is that participants may have believed that they would notice misinformation if any was presented, so they did not perceive the inoculation as relevant. This idea is supported by research showing that people are generally overconfident in their ability to detect misinformation and believe that other people will benefit more from inoculation than themselves [80,81]. These findings, combined with prior work showing that source discreditation can be effective at countering human-generated misinformation, could suggest that people believe that AI-generated misinformation is easier to identify and are therefore more confident in their ability to detect it than human-generated misinformation. Alternatively, people may have believed the arguments in the AI-generated article because they were consistent with their pre-existing beliefs [38] or relatively easy to process [20,82], despite being relatively distrustful of the AI source or AI-generated information generally. In line with this idea, research suggests that people may readily believe a non-credible source when the assertions are plausible or corroborated by others [59], and may engage in motivated reasoning when misinformation is consistent with their worldview [83–85]. Future research should investigate whether source discreditation is more effective on individuals who are less confident in their ability to detect misinformation, and whether the perceived believability of a misleading argument can moderate the effects of source discreditation.

The current research also provides further insight into when people are willing to trust AI-generated information. Specifically, we found that people were influenced by misleading information about trickle-down economics regardless of whether it was attributed to a human or AI source. This is in line with some prior research [86–88], but it may be a topic-dependent finding, as previous work has suggested that people are more willing to rely on AI in domains that are typically considered more objective, technical or analytical than in domains that are considered more emotional or moral [89–93]. The impact of source information may also vary depending on the strength of people’s pre-existing attitudes about the topic at hand. Generally, source credibility is likely to be less influential when people have strong pre-existing attitudes, as whether information is accepted or rejected in such cases may depend primarily on its compatibility with those attitudes [94]—noting that source-credibility evaluation itself can be influenced by pre-existing attitudes [95,96]. On the other hand, source information may be more influential when people make decisions based on conflicting information [97]. Caution should therefore be applied when generalizing the current findings to other topics.

Our findings nevertheless have implications for the use of AI labels on online platforms. By contrast to previous work suggesting that people perceive content labelled as AI-generated to be less accurate than the same information labelled as human-generated [25,26], our results indicate that AI labels do not necessarily increase scepticism, even when people are warned that AI systems can make mistakes. One important caveat is that the current study focused on reducing trust as a countermeasure to AI-generated misinformation. This differs from the goal of labelling AI-generated content, which may be to encourage people to carefully consider the veracity of AI-generated information without inducing general scepticism towards such information [98,99]. As the current study focused on misleading AI content, we could not examine how a pre-emptive source inoculation targeting generative AI systems may impact people’s responses to accurate AI-generated information [100–102]. Therefore, a clear target for future research is to examine whether labelling AI-generated information can improve people’s ability to discriminate between accurate and inaccurate information.

On a practical level, our findings support the recommendation that misinformation interventions include both source discreditation and content-focused correction [46]. We found that people continued to rely on misinformation to some extent after receiving a pre-emptive, source-focused inoculation or a retroactive, content-focused debunking, but misinformation reliance was eliminated when people received a combined intervention. These findings are consistent with previous research showing that source discreditation can significantly improve the effectiveness of other misinformation interventions (or vice versa), presumably because both the message and the messenger are targeted [46,74,103,104].

On a theoretical level, our results further highlight the need for models of continued misinformation influence to account for social variables, such as the perceived credibility of the misinformation source [49,53,58]. Specifically, the finding that source discreditation improves the effectiveness of retroactive corrections indicates that, although a failure to encode or remember corrective information likely contributes to continued influence effects, people’s evaluation of source credibility is a contributing factor, too. Source discreditation may also increase the effectiveness of content-focused interventions by reducing the psychological discomfort associated with processing corrections or by making corrections more memorable [48,105].

There are several limitations of the present study. First, trust in AI is known to be influenced by culture [106]. A worldwide survey, for example, found that people in developing countries tended to report higher levels of trust in AI systems and were more likely to believe that the benefits of AI outweighed the risks than people in Western countries [107]. In this study, we only recruited participants from the United States, where many people report having significant concerns about the use of AI systems. It is possible, therefore, that labelling content as AI-generated and/or discrediting AI systems may impact people’s responses to AI-generated information differently in other cultures. Future research could also examine whether amount of exposure to AI systems mediates the relationship between trust in AI and misinformation reliance across cultures.

Second, the current research only examined the impacts of AI-generated information presented outside an AI platform—as it may be encountered on news and social media sites—so participants did not engage with the generative AI system directly. Therefore, our findings cannot be used to draw conclusions about the direct impacts of AI-generated misinformation when people actively interact with an AI system (e.g. see [108]). In this space, future work may consider whether a source-focused inoculation may be more effective at reducing misinformation reliance when it includes an active component that provides people with direct experience of AI making mistakes. Although some research suggests that inoculation that requires active engagement (e.g. matching content with the persuasion technique used to mislead; [108–110]) can be effective at countering misinformation, no research—to our knowledge—has yet examined the effectiveness of such strategies for inoculating people against AI-generated misinformation. Additionally, given that people use heuristics to assess source credibility [111] and are sometimes more trusting of artificial agents that display humanlike features or behaviours [112–115], it may be fruitful for future research to examine whether targeting the more affective intuition dimension of trust and people’s tendency to anthropomorphize AI systems can influence trust in the information that these systems produce.

Finally, participants in the current study were only given the name of the alleged human author and therefore had little information to guide their credibility and veracity judgements. Thus, another question for future research may be whether people are more trusting of human-generated versus AI-generated information when the human author is associated with other credibility cues [52,116,117].

To conclude, the present study demonstrates that AI-generated misinformation has the potential to influence reasoning, regardless of whether people are aware of its AI source. Our findings suggest that the labels and generic disclaimers used by social media sites and generative AI platforms do not necessarily induce scepticism and may be of limited practical value for reducing reliance on AI-generated information. Retroactive debunking interventions that specifically counter the misinformation, however, are likely to be somewhat effective at reducing AI-misinformation reliance. Although a pre-emptive source discreditation alone was found to be insufficient to reduce reliance on AI-generated misinformation, the current research adds to the evidence that providing interventions that tackle both the message and the messenger may be crucial for eliminating misinformation effects.

## Data Availability

The data and materials for this study are available on the Open Science Framework (OSF): [118]. Supplementary material is available online [119].

## Footnotes

^1^
Although we had intuitions that the impact might be greater when the source was perceived to be human versus AI, we did not specify a direction for this hypothesis.

^2^
Based on age, gender, race and political orientation, matched to US census data.

^3^
There was no resistance from ChatGPT to generate a biased article, including fictional statements from fictional experts from fictional institutes, so no ‘prompt engineering’ was required.

^4^
While ChatGPT was also used to assist with the initial draft of this article, it was largely written by the senior author, not least to minimize the potential for it to be perceived as synthetically generated.

^5^
Experiments were run simultaneously; hypotheses therefore largely mirrored those of Experiment 1.

^6^
There was little resistance from ChatGPT to generate misleading information, so minimal ‘prompt engineering’ was required. ChatGPT was simply asked to ‘pretend’ it was in favour of trickle-down economics and that the request to use persuasion techniques including use of fake experts or evidence was for research purposes.
